# Direct-to-consumer advertising of success rates for medically assisted reproduction: a review of national clinic websites

**DOI:** 10.1136/bmjopen-2016-012218

**Published:** 2017-01-10

**Authors:** Jack Wilkinson, Andy Vail, Stephen A Roberts

**Affiliations:** 1 Centre for Biostatistics, School of Health Sciences, Faculty of Biology, Medicine and Health, Manchester Academic Health Science Centre (MAHSC), University of Manchester, Manchester, UK; 2 Research and Development, Salford Royal NHS Foundation Trust, Salford, UK; 3 Central Manchester University Hospital NHS Foundation Trust, Manchester Academic Health Science Centre (MAHSC), Manchester, UK

**Keywords:** in vitro fertilisation, advertising, success rates, assisted reproduction, live birth rate

## Abstract

**Objectives:**

To establish how medically assisted reproduction (MAR) clinics report success rates on their websites.

**Setting:**

Websites of private and NHS clinics offering in vitro fertilisation (IVF) in the UK.

**Participants:**

We identified clinics offering IVF using the Choose a Fertility Clinic facility on the website of the Human Fertilisation and Embryology Authority (HFEA). Of 81 clinics identified, a website could not be found for 2, leaving 79 for inclusion in the analysis.

**Primary and secondary outcome measures:**

Outcome measures reported by clinic websites. The numerator and denominator included in the outcome measure were of interest.

**Results:**

53 (67%) websites reported their performance using 51 different outcome measures. It was most common to report pregnancy (83% of these clinics) or live birth rates (51%). 31 different ways of reporting pregnancy and 9 different ways of reporting live birth were identified. 11 (21%) reported multiple birth or pregnancy rates. 1 clinic provided information on adverse events. It was usual for clinics to present results without relevant contextual information such as sample size, reporting period, the characteristics of patients and particular details of treatments.

**Conclusions:**

Many combinations of numerator and denominator are available for the purpose of reporting success rates for MAR. The range of reporting options available to clinics is further increased by the possibility of presenting results for subgroups of patients and for different time periods. Given the status of these websites as advertisements to patients, the risk of selective reporting is considerable. Binding guidance is required to ensure consistent, informative reporting.

**Strengths and limitations of this study**

- First review of outcome reporting by UK medically assisted reproduction clinic websites.
- Numerator and denominator of each reported item recorded, representing the variety of outcomes in use.
- Cross-sectional review, unable to comment on trends over time.
- Alternative methods of direct-to-consumer advertising (eg, social media) not considered.
- Method of categorising each clinic as NHS or private is imperfect.

## Introduction

Direct-to-consumer advertising of prescription drugs is permitted only in the USA and New Zealand. However, concerns that direct advertising drives demand for more expensive, rather than more effective, treatments do not extend to bans on direct advertising of other medical practices.

Questionnaires of subfertile patients have indicated that a majority make use of the internet to find information relating to their condition,^1^ ^2^ with a recent survey in Poland suggesting that 93% of respondents used online resources for this purpose.^3^ A key decision for any patient seeking treatment for subfertility is where to be treated, and it is expected that patients will take performance into account when choosing a fertility clinic. In practice, the reporting of success rates for medically assisted reproduction (MAR) is complicated by the complex, multistage nature of the treatments involved. Taking an in vitro fertilisation (IVF) cycle as an example, patients will typically undergo a period of ovarian stimulation before eggs are recovered and then fertilised. Some of the resulting embryos are then transferred to the uterus with the objectives of pregnancy and the subsequent birth of a healthy child. Failure may occur at each step in this sequence, so that a considerable variety of numerators (such as pregnancy or live birth) and denominators (such as started cycles, transfer procedures or egg collections) may be used.^4^ Furthermore, since patients typically undertake multiple attempts at treatment, there is the option to report outcomes in a cumulative fashion. For example, live birth rates could be reported following several stimulation or transfer procedures. Consequently, the matter of how MAR success rates should be reported has been extensively discussed in the literature^5–12^ and has featured in a recent consultation process (‘Information for Quality’) by the Human Fertilisation and Embryology Authority (HFEA).^13^ There is also the question of how to report adverse consequences of treatment. In particular, given the HFEA policy of reducing the number of twin births arising from MAR, the reporting of multiple pregnancy rates requires attention.

In addition to informing clinic selection, reported outcomes may also be used by patients trying to understand their own chances of success. At present, HFEA present success rates in the form of live birth per cycle started and live birth per embryo transferred on its online Choose a Fertility Clinic facility, a new version of which is currently being tested.^14^ This information is presented separately for treatments involving fresh and frozen embryos, for patients using their own or using donated gametes and for different age groups. Furthermore, the particular treatment variants included in the results, the sample sizes and the reporting period are all presented. In principle, the provision of this contextual information makes it possible for patients to identify relevant results and to consider these when making decisions about whether and where to start treatment. Although HFEA provide standardised reporting of success rates, no such standardisation is imposed on clinics' own websites. In the light of this, the consistency and clarity of online reporting is of material interest.

In order to investigate the standards of reporting of MAR success rates, we conducted a national review of MAR clinic websites. Our aim was to identify the outcomes in use by clinics and to examine whether results were presented in a consumer-friendly manner.

## Methods

### Identification of websites

We restricted our focus specifically to clinics offering assisted reproductive technology (ART), although we extracted information about other MAR treatments, such as intrauterine insemination (IUI), which would not be considered ART.^15^ An initial search was made between 26 January 2015 and 29 January 2015 on the HFEA Choose a Fertility Clinic facility,^14^ using the search options ‘both’ for the field ‘funding for patients’ and ‘IVF’ for ‘treatments offered’. An earlier scoping exercise had suggested that no clinic offered intracytoplasmic sperm injection but not in vitro fertilisation (IVF). This search was performed for each of the 12 ‘regions’ listed by HFEA. The website addresses of each clinic were recorded. Where the website listed by HFEA was inactive, or where no website was listed, the correct address was obtained via Google searching. It became apparent that this method had not produced a complete list of clinics. Accordingly, a further search was made using the A to Z listings on the HFEA website on 4 February 2015 and 5 February 2015. Any clinics offering IVF that were not identified during the initial search were added to the data set. Again, missing or defunct website addresses were updated by searching on Google. As a final check, the initial search was repeated on 5 February 2015 with the ‘funding for patients’ field replaced by each of ‘private’ and ‘nhs’. Although this revealed clinics that had not been identified during the initial search, it did not reveal any clinics that had not been identified after the A to Z search. Where multiple clinics shared a website, we used the centre-specific results for analysis, so that the clinic was the unit of analysis.

### Data extraction

Data were extracted at the clinic level and for each reported result on the clinic's website. At the clinic level, we recorded the type of patients treated (NHS, private or both), whether or not an NHS logo was displayed on the front page, whether or not patient testimonials were used, and if so whether or not these were featured on the front page, whether selection policies relating to body mass index (BMI), age, number of previous attempts and smoking status were reported and whether the website reported success rates. At the result level, we extracted the numerator and denominator used, together with the definition of the numerator if provided. We further extracted the corresponding patient and cycle characteristics for the reported item, including patient age range, treatments included, whether donor gametes were included, whether fresh or frozen cycles were included (for treatments other than IUI), the sample size, the reporting period as well as the number of cancellations and incomplete treatments. For each of these, we recorded instances where the required information could not be identified from the presented results.

### Statistical analysis

We summarised the characteristics of the clinic websites, tabulating the numerators and denominators in use within five categories: pregnancy, live birth, multiple births, preclinical outcomes and adverse events. We calculated the proportion of clinics where results were presented in such a way so that each of age range, included treatments, inclusion of donor gametes, inclusion of fresh/frozen cycles, sample size, number of abandoned treatments and reporting period could not be identified. We were particularly interested in whether or not clinics achieved the standard of reporting adopted by HFEA. To this end, we calculated the proportion of websites reporting the outcomes ‘live birth per cycle started’ and ‘live birth per embryo transferred’ together with all of the relevant contextual information (ie, all of the factors listed above with the exception of the number of abandoned treatments, as these cycles are included as failures in rates reported per cycle started).

We calculated the proportion of websites for which patient selection policies were not stated. Finally, we made a tentative comparison between NHS and private clinics in relation to standards of reporting, although we did not consider statistical inference to be particularly meaningful in relation to this.

## Results

### Characteristics of clinics

The search identified 81 clinics in the UK. Of the 81 clinics identified, a website could not be found for 2, leaving 79 for the present analysis. Fifty-three (67%) reported outcomes. Among those reporting outcomes, there was considerable variation in the number reported; the median (range) was 36 (1–127). Sixty-two (78%) stated that they treated NHS and private patients, 4 (5%) described themselves as treating NHS patients only and 13 (16%) stated that they exclusively treated private patients. Twenty-three (29%) displayed an NHS logo on the front page. Forty-nine (62%) of the websites featured patient testimonials, of which 23 (47%) featured these on the front page.

### Reported outcomes

**Figure 1:**
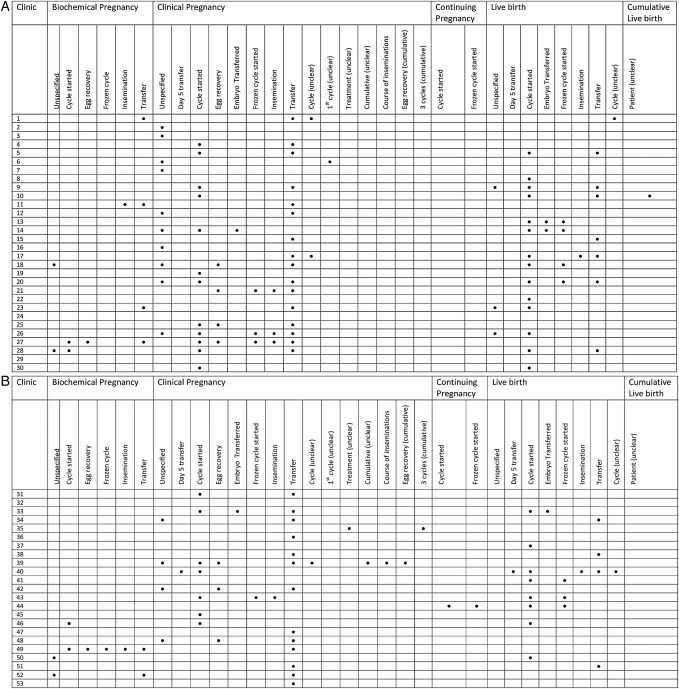
(A) Distribution of clinical outcome measures reported on medically assisted reproduction (MAR) clinic websites. The denominator used is displayed for each numerator. (B) Distribution of clinical outcome measures reported on MAR clinic websites. The denominator used is displayed for each numerator.

A total of 54 different outcome measures were identified during the review. The distribution of clinical outcome measures across the clinics is shown in figure 1A, B.

#### Pregnancy outcomes

Thirty-three different ways of reporting pregnancy were identified (table 1). The majority (81%) of clinics reported clinical pregnancy rates, with most (55%) websites reporting these per transfer procedure. A substantial proportion (36%), although fewer than half, reported clinical pregnancy per cycle started. Notably, around one in four websites reported clinical pregnancy rates without specifying the denominator. Just under a fifth (19%) of websites presented biochemical pregnancy rates, and these were most commonly reported per transfer (11%), per cycle started (8%) or without specifying the denominator (8%). Over a fifth (21%) of clinics presented pregnancy rates without explaining what was meant by ‘pregnancy’, with 15% also leaving the denominator unspecified. Reporting of cumulative outcomes across multiple transfers or inseminations was sparse, with no site reporting biochemical pregnancies and only a small number reporting clinical pregnancy rates cumulatively. One site reported continuing pregnancy rates. The median reporting period for pregnancy outcomes was 1 year; this ranged from 3 months to 10 years. Just three clinics reported up-to-date clinical pregnancy rates (covering the end of 2014). Twenty clinics (47% of those reporting clinical pregnancy) reported clinical pregnancy rates for multiple time periods, giving some indication of trends in performance.

**Table 1:** Reported pregnancy outcomes

Outcome numerator	Denominator	Number (%) of clinics reporting item
Biochemical pregnancy		10 (19% of clinics)
	Unspecified denominator	4 (8)
	Per cycle started	4 (8)
	Per egg recovery	2 (4)
	Per frozen cycle	1 (2)
	Per insemination (IUI)	2 (4)
	Per transfer procedure	6 (11)
Clinical pregnancy		43 (81% of clinics)
	Unspecified denominator	14 (26)
	Per day 5 transfer	1 (2)
	Per cycle started	19 (36)
	Per egg recovery	7 (13)
	Per embryo transferred	2 (4)
	Per frozen cycle started	4 (8)
	Per insemination (IUI)	4 (8)
	Per transfer procedure	29 (55)
	Per cycle (ambiguous)	3 (6)
	Per first cycle (ambiguous)	1 (2)
	Per treatment (ambiguous)	1 (2)
	Unspecified denominator (cumulative)	1 (2)
	Per course of inseminations (cumulative, IUI)	1 (2)
	Per egg collection (cumulative)	1 (2)
	Per three cycles (cumulative)	1 (2)
Pregnancy (unspecified)		11 (21% of clinics)
	Per patient (cumulative)	1 (2)
	Per three cycles (cumulative)	1 (2)
	Unspecified denominator	8 (15)
	Per day 5 transfer	1 (2)
	Per cycle started	3 (6)
	Per frozen cycle started	1 (2)
	Per insemination (IUI)	1 (2)
	Per transfer procedure	2 (4)
	Per cycle (ambiguous)	2 (4)
Singleton pregnancy		1 (2)
	Unspecified denominator	1 (2)
Continuing pregnancy		1 (2)
	Per cycle started	1 (2)
	Per frozen cycle started	1 (2)

Number (%) of clinics reporting each outcome.

#### Live birth outcomes

Just over half (51%) of the clinics reported live birth rates, with 9 different live birth outcomes identified (table 2). In contrast to pregnancy outcomes, it was most common to report live birth per cycle started (42% of clinics) as opposed to per transfer procedure (21%), perhaps reflecting the use of live birth as a patient-orientated outcome. A small number (6%) reported live birth per embryo transferred, although it could not be ascertained whether this was genuinely what was being reported or if this phrase had been used erroneously. A small number of websites (6%) reported live birth rate without defining the denominator. Just one website reported live birth rates cumulatively. These were reported ‘per patient’, although it was unclear at what point patients' data were censored. This website also reported the average number of cycles for those who achieved live birth (1.6), although this does not convey information about the expected number of cycles required to a patient faced with the decision of whether or not to start IVF.

**Table 2:** Reported live birth outcomes

Outcome numerator	Denominator	Number (%) of clinics reporting item
Live birth		27 (51% of clinics)
	Unspecified denominator	3 (6)
	Per day 5 transfer	1 (2)
	Per cycle started	22 (42)
	Per embryo transferred	3 (6)
	Per frozen cycle started	7 (13)
	Per insemination (IUI)	2 (4)
	Per transfer procedure	11 (21)
	Per cycle (ambiguous)	2 (4)
Cumulative live birth		1 (2% of clinics)
	Per patient	1 (2)

Number (%) of clinics reporting each outcome.

Only one clinic reported live birth per cycle started in such a way that patient age, sample size, included treatments, inclusion of fresh and/or frozen cycles, inclusion of donor cycles and reporting period were all clear. Nine (17%) clinics reported live birth per cycle started with each of age, sample size and period. Live birth rates were reported for a median time period of 1 year. However, this ranged from 3 months to 14 years. It is unclear how valid live birth rates can be reported for such short periods (the 3-month rates come from one clinic, the only one reporting live birth for a period of <1 year). Just three clinics reported live birth rates that were up to date (results from 2013 would have been available at the time of this review), although one of these stated that the results covered the whole of 2014, which is not possible, given the follow-up period required to establish live birth. Ten clinics (37% of clinics reporting live birth rates) reported live birth rates for multiple calendar periods, providing evidence of trends in performance.

#### Multiple births

Eleven (21%) clinics reported information on multiple birth or pregnancies. Six (11%) clinics reported multiple birth rates. These were reported per live birth (two clinics), per cycle (one clinic) or without specifying the denominator (three clinics). Eight (15%) clinics reported multiple clinical pregnancy or multiple pregnancy rates. The denominator was either unspecified (four clinics) or per pregnancy (four clinics).

#### Preclinical outcomes

Just two clinics reported preclinical outcomes. Blastocyst achievement (with no denominator), implantation (no denominator) and transfer achieved per frozen cycle each appeared on one site.

#### Adverse events

Only one clinic reported adverse outcomes. Ectopic pregnancy and miscarriage were reported, although denominators were not specified.

### Reporting of contextual information

Of the 53 clinics reporting outcomes, 14 (26%) presented (at least some) outcomes without specifying the age of the patients, 38 (72%) presented outcomes without specifying the treatments, 38 (72%) presented outcomes without specifying the sample size and 12 (23%) presented outcomes without specifying the period these related to. Forty-eight (91%) presented outcomes for which it was unclear whether or not donor gametes were used. Forty-two (84%) presented outcomes for non-IUI treatments where it was unclear whether included cycles were fresh, frozen or both fresh and frozen. Fifty (94%) presented outcomes without specifying how many patients did not complete the treatment.

Inclusion/exclusion criteria were not consistently reported. Criteria relating to BMI could not be found for 64 (82%) of the websites, to age for 67 (85%) of the websites, to previous attempts for any website or to smoking status for 94% of websites. Sixty-three (80%) sites did not appear to provide criteria relating to any of these characteristics.

### Comparison of NHS and private clinics

A higher proportion of NHS clinics compared with private centres reported age (89% vs 66%), sample size (50% vs 17%), use of donor gametes (17% vs 6%), use of fresh or frozen embryos (24% vs 12%, excluding IUI treatments) and number of abandoned treatments (17% vs 0%) for all outcomes. The proportion of NHS (28%) and private (29%) centres specifying the treatments involved for all reported results was similar. More private clinics (80%) than NHS clinics (72%) reported the date range for all outcomes.

## Discussion

The present review confirms inconsistency in clarity and coverage when advertising clinic success rates with only one meeting HFEA's own standards. In addition to selecting from a number of numerators and denominators, clinics may also report results for different combinations of treatments, fresh and frozen cycles, donor and non-donor cycles and for different calendar periods. The large number of numerators and denominators in use constitutes an obstacle to consumer-friendly reporting, as patients may struggle to understand subtle differences in outcome definitions and may be misled into making comparisons between centres on the basis of incommensurable results.^16^

Allowance of open reporting without binding guidelines carries a high risk of selective reporting; there is scope for clinics to construct more favourable outcomes using the variety of building blocks available. These points were highlighted by direct comparisons with other clinics using a ‘league table’ presentation on 9 (11%) of the 79 websites. League tables are known to be problematic due to differences in patient characteristics and imprecision in the results used to create them.^17^ In addition to choices relating to outcome definition, league tables additionally allow clinics to select which other centres to include. These tables were invariably constructed, so that the comparison was favourable to the reporting clinic. In one case, two websites used the outcome ‘live birth per cycle started’ as the basis for a comparative table. Despite displaying results for overlapping (but not identical) time periods, one table indicated a considerable advantage of the reporting clinic over its competitor, while the other indicated that the performance of both clinics was comparable. The results used in both tables could not be called inaccurate.

The review raises concerns relating to clarity of reported results, with implications for patient usability. Current reporting trends are to present results in such a way so that the included treatments and inclusion or exclusion of frozen or donor gametes are often unclear. Given the multiplicity of relevant factors, a plausible rationale for these practices is to maintain simplicity. Complexity does represent a concern, as stakeholders may have difficulty interpreting conditional risk presented in the form of frequencies and percentages (eg, ref. 18). However, by obscuring the particular patients and treatments for which results are presented, omission of such relevant information may in fact serve to obfuscate what is being reported. It was also common to report outcomes without sample sizes and without indicating the number of cycle cancellations or otherwise incomplete treatments, with implications for understanding the precision and the prognostic relevance of the results, respectively.

An emphasis on pregnancy was evident, with pregnancy outcomes representing the most common way to report success. The most common denominator used when reporting pregnancies was per transfer procedure. Considerably fewer clinics reported live birth rates. In contrast to pregnancy results, it was most common for these to be reported per cycle started. It has been argued that live birth is the most relevant measure of success of MAR to patients owing to the fact that this is the goal of any initiated treatment^4^ ^10^ ^12^ ^19–21^ and that it is more informative to include all patients starting treatment by counting events per cycle started.^4^ ^10^ Given that patients often undergo multiple attempts as part of their treatment, a case may be made for success rates to be presented cumulatively across some set time period or number of cycles.^4^ ^22–31^ We found very few instances of this in the present study. This may be due to the practical challenges of calculating these cumulative rates and the need for a lengthy delay in reporting. HFEA have indicated that they will include cumulative live birth rates on their own website in future however. It is important to recognise that different outcomes may be suitable for different purposes, so that no single measure of success can be recommended. One proposal is that, whereas live birth per cycle started or per course of treatment may hold greater prognostic value, ongoing pregnancy may be more relevant for clinic performance evaluation.^8^ A clear concern when deciding on an appropriate performance measure is the impact that this may have on clinic behaviour. Clinics compete for patients, who are encouraged to consider performance when choosing a clinic.^32^ There is therefore an incentive to potentially modify the treatment delivered in order to optimise a particular performance indicator. This sort of gaming can lead to perverse behaviour which might not guarantee the best outcomes from a patient perspective.^33^ This could manifest, for example, by clinics imposing tougher selection criteria, which we found to be sparsely reported.^34^ Without clearly presented selection policies, it is impossible to understand how much of a clinic's performance to attribute to treatment effectiveness and how much to the reproductive competence of their patients. We acknowledge that some centres may not have strict selection criteria, instead offering treatment to anyone who is able to pay. Nevertheless, it would be useful if these clinics reported that their results were based on relatively unselected cohorts. The desire to manipulate the behaviour of clinics to the advantage of patients motivates the proposal of live birth per embryo transferred as a measure of success, in order to encourage the transfer of fewer embryos at each attempt and to thereby reduce the incidence of multiple births.^5^ On these grounds, HFEA plan to make live birth per embryo transferred the headline figure on their own website following their Information for Quality consultation.^13^ However, such a proposal introduces further complication as multiple embryos are not statistically independent.

Policies to reduce twin rates are ubiquitous outside the USA, and numbers of multiple births represent an important measure of clinic performance. Despite this, only 11 sites reported on multiple birth or pregnancy rates. Only one site reported on other adverse events. In the USA, omission of information relating to side effects has been noted as a characteristic of direct-to-consumer advertising of prescription drugs, with a substantial proportion of regulatory letters sent to manufacturers by the Food and Drug Administration (FDA) citing advertisements for minimisation of risks.^35^ It has been suggested that spending on direct-to-consumer advertising in the USA increased drastically following changes to FDA regulations in 1997 that allowed manufacturers to advertise products without explicitly listing side effects,^36^ although there is some evidence that the trend for increased spending actually preceded these changes.^37^ In the present study, reporting of cancellations and abandoned treatments was also scanty, so that the actual chances of success for patients starting treatment could often not be discerned.

Our findings add to a body of literature highlighting the difficulty of reporting MAR outcomes in a consumer-friendly way. A 2007 review assessed US clinic websites according to the American Society for Reproductive Medicine/Society for Assisted Reproductive Technology guidelines, and found generally low compliance.^38^ An earlier assessment of US clinic websites suggested generally low quality according to a scoring system based on American Medical Association internet health information guidelines,^39^ although the methodology of the study has been queried, given the status of these websites as advertisements.^40^ ^41^ In the UK, a 2008 review of UK websites providing information on infertility found the quality of information to be variable, with particular concerns about accuracy.^42^ Quality control of data is essential for reliable performance monitoring.^33^ At present, there is no way to guarantee the quality or accuracy of data presented on clinic websites.

The present study would appear to represent the first review of outcome reporting by UK MAR clinic websites. Strengths of the study include the extraction of item-level data, allowing the variety of outcomes in use by UK clinics to be presented. Limitations of the study should be noted. In particular, this review was cross-sectional, meaning that we are unable to comment on reporting trends over time. We have also not considered alternative ways in which clinics use the internet to communicate results to patients, such as social media. Our comparison of NHS and private clinics is also tentative; we used the presence or absence of the NHS logo on the front page of the site to distinguish NHS from private centres, with one exception (a private clinic where the logo was clearly used to illustrate an existing NHS contract). This method is obviously imperfect, and while we believe that we managed to correctly categorise clinics, it is possible that some misclassification occurred. With these limitations in mind, we conclude that self-regulation does not appear to guarantee clear, patient-friendly reporting of outcomes.

Our intention is not accusatory; the matter of how to report MAR outcomes is complex and we expect that many clinics present their success rates in good faith. There are clear parallels to ongoing discussions about the presentation of online information in other areas, such as cosmetic procedures (eg, ref. 43) or complementary medicine (eg, ref. 44). There is a tension between ‘open reporting’ in the interests of transparency and ‘direct to consumer advertising’, particularly for private providers. One method to address this would be binding guidance for consistent content in reporting results. Another would be an outright ban on direct advertising of MAR.
